# Sex Reporting in Preclinical Microbiological and Immunological Research

**DOI:** 10.1128/mBio.01868-17

**Published:** 2017-11-14

**Authors:** Tanvi Potluri, Kyrra Engle, Ashley L. Fink, Landon G. vom Steeg, Sabra L. Klein

**Affiliations:** W. Harry Feinstone Department of Molecular Microbiology and Immunology, Johns Hopkins Bloomberg School of Public Health, Baltimore, Maryland, USA

**Keywords:** immunology, microbiology, sex reporting

## Abstract

Both sex (i.e., biological construct of male and female) and gender (i.e., social construct of masculine and feminine) impact the pathogenesis of diseases, including those caused by microbial infections. Following the 2015 NIH policy for consideration of sex as a biological variable in preclinical research, in 2018, authors of papers published in primary-research American Society for Microbiology (ASM) journals will be asked to report the sex of the research subjects and animals and of materials derived directly from them. To address the need for sex reporting in ASM journals, we systematically reviewed 2,928 primary-research articles published in six primary-research ASM journals (*Antimicrobial Agents and Chemotherapy*, *Clinical and Vaccine Immunology*, *Infection and Immunity*, *Journal of Bacteriology*, *Journal of Virology*, and *mBio*) in 2016. Approximately 37% of animal studies and 9% of primary cell culture papers published in 2016 would have been affected by the new sex-reporting policy. For animal studies (i.e., studies with any nonhuman vertebrate hosts), most published papers either did not report the sex of the animals or used only female animals, and a minority used only males or both sexes. For published studies using primary cells from diverse animal species (i.e., humans and nonhuman vertebrates), almost all studies failed to report the sex of donors from which the cells were isolated. We believe that reporting the sex of animals and even of the donors of derived cells could improve the rigor and reproducibility of research conducted in microbiology and immunology and published in ASM journals.

## GUEST EDITORIAL

Basic scientists, clinicians, and epidemiologists alike often use the terms “sex” and “gender” interchangeably in microbiology and immunology research, which is incorrect because these terms refer to different aspects of biology and behavior. The term “sex” is a biological construct that defines males and females by the basic organization of chromosomes, reproductive organs, and circulating sex steroid hormone concentrations. Gender is a social construct that refers to the attitudes and behaviors that influence the roles and activities, including education, occupation, and health-seeking behaviors, of men and women (1). Both sex and gender can impact the pathogenesis of infectious diseases by influencing the biology and behaviors of males and females differentially.

**FIG 1:**
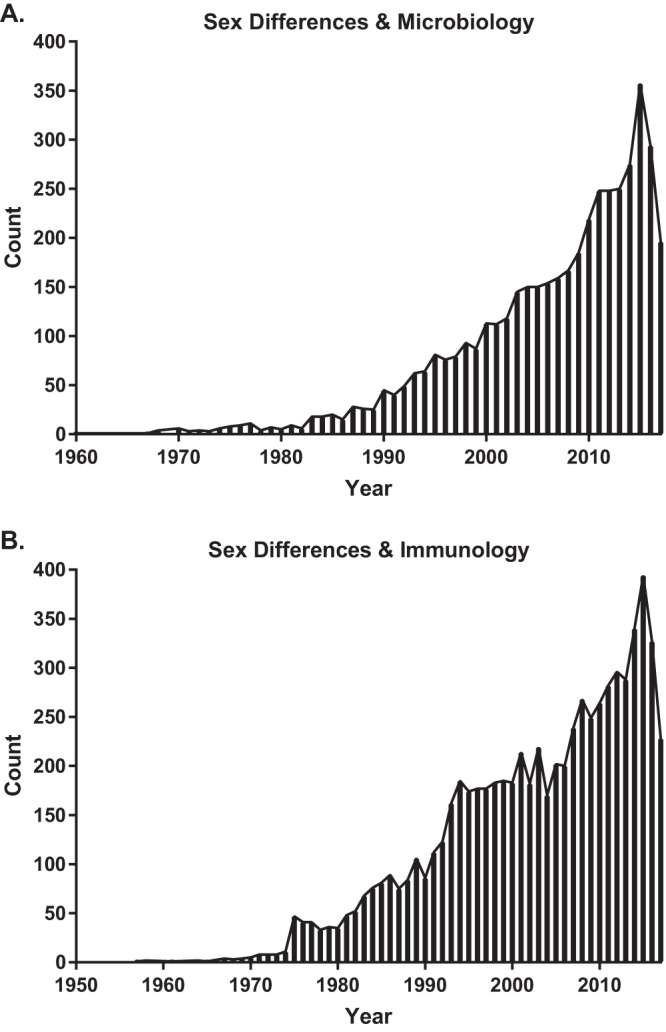
The number of published papers listed in PubMed pertaining to sex differences in microbiology and immunology. On 26 September 2017, PubMed search results for “sex differences” and “microbiology” yielded a total of 4,456 papers published between 1960 and 2017 (A) and search results for “sex differences” and “immunology” yielded a total of 7,086 papers that were published between 1957 and 2017 (B). For each search, there was an increase (i.e., >100 papers/year) in the 1990s. Since the 1990s, there has been a steady increase in the number of published papers in microbiology and immunology relevant to sex-difference research.

Published reports of differences between males and females in diagnosis, presentation, and pathogenesis following infection with diverse microbial pathogens are rapidly increasing in number (Fig. 1A). During the 2014 Ebola virus outbreak in West Africa, for example, males reportedly had a longer duration of hospitalization and a higher case fatality rate than females (2). Historic studies of HIV (i.e., prior to the prophylactic use of antiretrovirus therapies) revealed that during acute infection, women had over 40% less HIV RNA in circulation than men. The prevalence of serum hepatitis B virus (HBV) surface antigen, HBV DNA titers, and the rates of development of hepatocellular carcinoma are higher in men than women (3). In most countries, two times more tuberculosis notifications are received for men than women (4). In tropical and subtropical countries, 80% of patients with amoebic liver abscess (including travelers to those countries), caused by the protozoan parasite *Entamoeba histolytica*, are men (5). Among immunocompromised patients, clinical cryptococcosis is 10 times more frequent in men than women (6). As a general rule, males are more susceptible to infection with diverse pathogens than females, but the underlying causes for greater susceptibility in males are diverse and in many cases not known.

In addition to differences in the pathogenesis, prognosis, and outcome of infectious diseases, there are profound differences between the sexes in immune responses that control as well as contribute to the development of disease (Fig. 1B). Females of diverse species, including humans, typically develop higher innate, cell-mediated, and humoral immune responses than males, which can affect vaccine efficacy, reduce pathogen load, and accelerate pathogen clearance but can be detrimental by causing immune-mediated pathology as well as autoimmune or inflammatory diseases (7). Despite significant immunologic differences between the sexes, in 2009, an analysis of diverse biomedical fields revealed that a majority of published studies in immunology, including those published in *Infection and Immunity*, either do not report the sex of their subjects or do not disaggregate and analyze data by sex (8). The status quo is to assume that the sexes do not differ (8), which has hindered our understanding of the pathogenesis of infectious diseases and the underlying immunologic mechanisms.

To begin to remedy this situation, in 2015, the National Institutes of Health (NIH) rolled out policy changes, including the requirement of considering “sex as a biological variable” in preclinical research (9). This policy change has been met with mixed reactions, including concerns about doubling of animal numbers in preclinical experiments. Many commentaries (10–12) have mitigated this concern as the policy requests adequate consideration of sex in all experiments, without the need for statistical power to detect all differences between males and females. The policy states that, unless justified, one-half of all subjects should be female and the remainder should be male. The policy changes at NIH, including consideration of sex as a biological variable, are aimed at addressing one of the biggest problems facing the scientific community to date—rigor and reproducibility.

In addition to NIH policy changes, several journals have implemented policies to promote reporting of the sex of animals and primary cells in preclinical research (https://genderedinnovations.stanford.edu/sex-and-gender-analysis-policies-peer-reviewed-journals.html), with guidelines outlined in an Institute of Medicine report (13) and the SAGER (Sex and Gender Equity in Research) guidelines (14). Adding to the list of journals implementing sex-reporting policies, the American Society for Microbiology (ASM) will be modifying their instructions to authors in 2018 to highlight the requirement that the authors of papers published in primary-research ASM journals report the sex of the research subjects and animals and of materials derived directly from them (e.g., primary cells or clinical samples). This information should be included in Materials and Methods or in Results. There are exceptions, of course, including cases where microbiological samples have not been and cannot be readily deidentified for either sex or gender and immortalized cells, which due to sex chromosomal abnormalities, are excluded from sex reporting.

**FIG 2:**
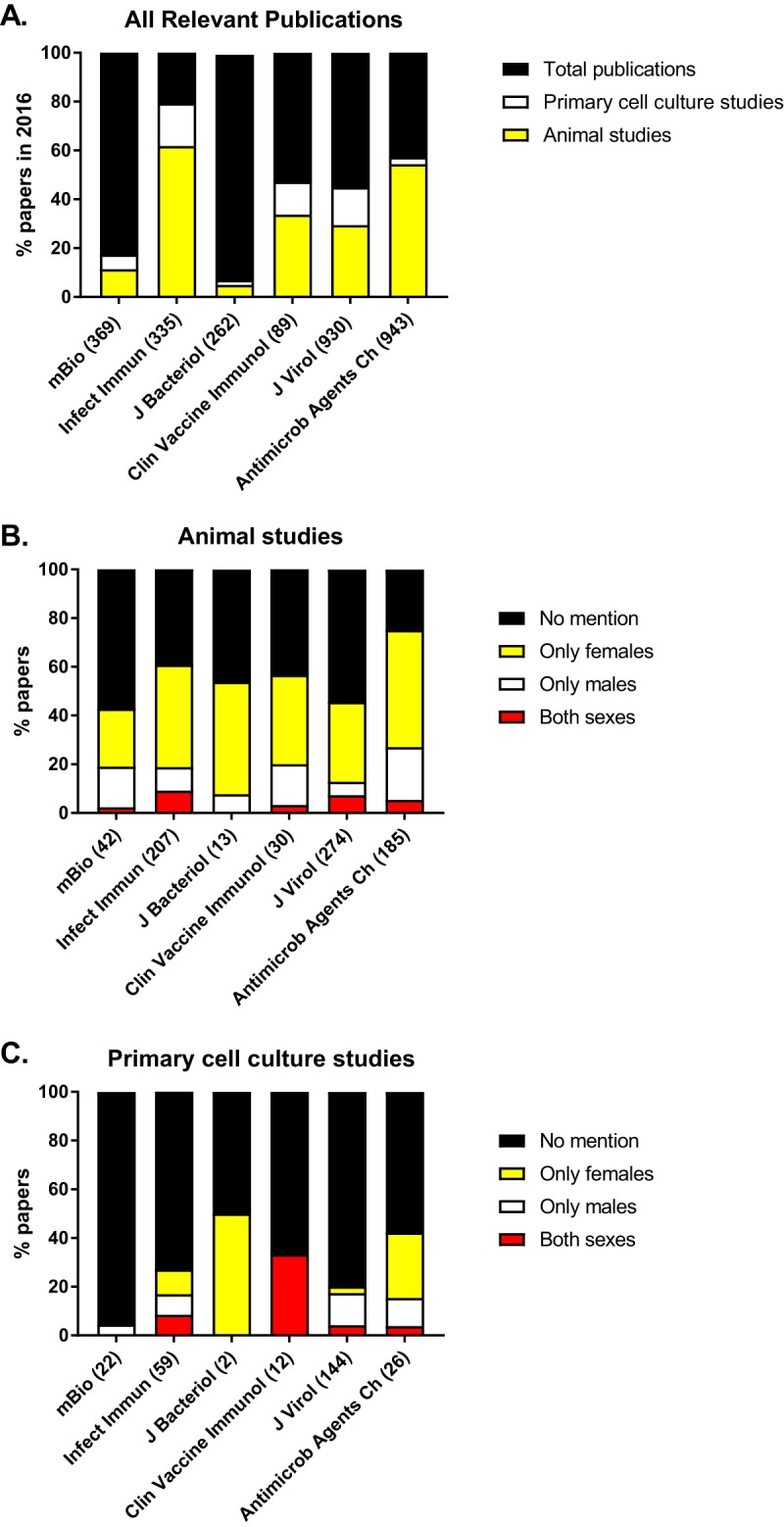
The proportion of papers published in 2016 in ASM journals that would be affected by a sex-reporting policy (A) and that report the sex of their animals (B) or primary cell cultures (C). The proportion of papers published in each of six ASM journals (i.e., *Antimicrobial Agents and Chemotherapy*, *Clinical and Vaccine Immunology*, *Infection and Immunity*, *Journal of Bacteriology*, *Journal of Virology*, and *mBio*) describing nonhuman animal research or the use of primary cell cultures from either humans (e.g., peripheral blood mononuclear cells stimulated and tested *in vitro*) or nonhuman animals (e.g., bone marrow-derived cells differentiated, stimulated, and tested *in vitro*) was determined (A) and then evaluated for whether the papers reported the sex of their animals (B) or the donors of their cells (C) and, if so, whether only males, only females, or both sexes were used. Numbers in parentheses represent the total number of papers in each category.

To gain a better understanding of the need for sex reporting in ASM journals, we systematically reviewed 2,928 primary-research articles published in six primary-research ASM journals (*Antimicrobial Agents and Chemotherapy*, *Clinical and Vaccine Immunology*, *Infection and Immunity*, *Journal of Bacteriology*, *Journal of Virology*, and *mBio*) in 2016. We collected data from 2016 because this represented all papers published in the selected ASM journals within a year after implementation of the NIH policy changes pertaining to sex as a biological variable. Primary-research articles were first categorized by whether there was use of vertebrate animals or primary cell cultures and then by whether the sex of the subjects was reported. If sex was reported, then articles were stratified based on whether only males, only females, or both sexes were used. Reviews, editorials, and other non-primary-research articles were excluded from our analysis. We first determined the proportion of published papers in each of the six selected ASM journals that would be affected by a sex-reporting policy change for preclinical research. Our results indicate that of the primary-research papers published in these six primary-research ASM journals in 2016, approximately 37% of animal studies and 9% of primary cell culture papers could have been affected by the new sex-reporting policy. It is noteworthy, however, that for some journals, e.g., *Infection and Immunity*, 80% of the published papers would be affected by this policy, whereas for other journals, e.g., *Journal of Bacteriology*, <10% of published papers involved the use of vertebrate animals or primary cells (Fig. 2A). For each of the six primary-research ASM journals, we then analyzed the papers that reported the use of vertebrate animals and primary cells to determine the proportion that reported the sex of the subjects and, if the sex was reported, the proportion that used only females, only males, or both sexes. Our analysis revealed that for animal studies (i.e., studies that used any nonhuman vertebrate host), most published papers either did not report the sex of the animals or used only female animals (Fig. 2B). A minority of published papers in these selected journals involving animals used only males or both sexes. For published studies using primary cells from diverse animal species (i.e., humans and nonhuman vertebrates), almost all studies failed to report the sex of donors from which the cells were isolated (Fig. 2C). Considering the results of our analysis of papers published in ASM journals in the context that an estimated 51 to 89% of animal studies in the biomedical sciences are not reproducible (15), we believe that reporting the sex of animals and even of the donors of derived cells could improve the rigor and reproducibility of research conducted in microbiology and immunology and published in ASM journals.
